# A CAD-to-Simulink framework for evaluating impingement-free motion in reverse total shoulder arthroplasty

**DOI:** 10.1177/09544119261471200

**Published:** 2026-07-23

**Authors:** Mercy Ombogo, John Medley, G. Daniel G. Langohr, Naveen Chandrashekar

**Affiliations:** 1 Department of Mechanical and Mechatronics Engineering, University of Waterloo, ON, Canada; 2 Department of Mechanical and Materials Engineering, Western University, London, ON, Canada

**Keywords:** reverse total shoulder arthroplasty, impingement-free range of motion assessment, contact detection, MATLAB, Simulink, neck-shaft angle, kinematic modeling, implant design optimization

## Abstract

Accurate characterization of impingement-free range of motion (ROM) following reverse total shoulder arthroplasty (RTSA) is essential for implant design and surgical planning. Existing computational methods often rely on mesh-overlap or clearance-based estimations, which can limit geometric accuracy. This study introduces and validates a CAD-to-Simulink computational pipeline that predicts impingement-free ROM using a high-resolution, point-cloud-based collision detection approach. Computer models of shoulder bony anatomy and generic RTSA implant components were assembled in SolidWorks and exported to MATLAB. Automated scripts executed humeral motion sweeps across planes of elevation. Point clouds were seeded on the acromion, coracoid, and scapular neck and used to detect impingement when penetration depth ≥1 mm. Three neck-shaft angles (155°, 145°, 135°) were evaluated. The 155° configuration was validated experimentally using additive manufactured monoblocs mounted on a custom-made frame under a 15 N compressive load. Sensitivity analyses examined the influence of point cloud density and spacing on impingement detection accuracy. Computational results demonstrated that decreasing the neck-shaft angle from 155° to 135° increased impingement-free ROM across evaluated planes, with improvements of up to 23°. This gain was attributed to delayed inferior impingement during adduction. Experimental validation closely aligned with predictions, showing impingement at 61.4° (superior) and 20.9° (inferior), compared to predicted values of 61.4° and 21.0°. Sensitivity analyses highlighted the importance of point cloud placement along key scapular regions. The CAD-to-Simulink framework provides a validated, reproducible, Finite Element Method independent prediction of impingement-free ROM, providing a kinematically accurate tool to optimize implant geometry and surgical positioning in RTSA.

## Introduction

Reverse total shoulder arthroplasty (RTSA) is an established treatment for cuff deficient shoulders, yet optimizing implant geometry and surgical positioning to maximize postoperative functional range of motion (ROM) remains challenging.^1–6^ Impingement has emerged as the primary constraint limiting functional ROM following RTSA. Variations in implant design parameters, such as neck shaft angle, cup depth, lateralization, center of rotation location, and inferior glenosphere overhang have been shown to significantly influence impingement limits.^1,3–5,7–12^

Prior biomechanical studies have demonstrated linear relationships between implant design parameters and postoperative ROM. For instance, Roche et al.^5^ reported the existence of a linear relationship between specific design parameters, glenosphere geometry, humeral liner geometry, neck shaft angle, and postoperative functional ROM. Similarly, Zitnay et al.^4^ demonstrated that humeral distalization improves active postoperative ROM using varying custom humeral polymer inserts. Consistent with these findings, Cogan et al.^3^ further showed that reducing the neck-shaft angle from 155° to 135° and lateralizing the glenosphere enhanced functional motion while minimizing scapular notching.

Central to understanding how design variations affect postoperative ROM are experimental and computational biomechanical investigations. In vitro investigations, such as cadaveric studies, provide crucial validation of implant performance under physiological conditions and allow for direct observation of joint mechanics.^4,6,13,14^ However, despite their importance, experimental studies are inherently constrained by inter-specimen variability, small sample sizes, and methodological challenges from extensive tissue resection, even in large, highly controlled investigations. For instance, Berhouet et al.^6^ examined 40 cadaveric shoulders to evaluate the effects of prosthesis design and implantation technique on ROM. Despite this scale and methodological rigor, variability in scapular morphology and the limited ability to assess certain motions after detachment of the shoulder from the torso restricted their capacity to isolate the effects of implant geometry. Consequently, it becomes difficult to attribute ROM differences exclusively to implant geometry rather than specimen-specific factors in such studies.

Conversely, computational approaches have emerged as preferred alternatives for evaluating design modifications under controlled and repeatable conditions.^15,16^ These methods allow for systematic exploration of parameter interdependencies across multiple motion trajectories, thereby revealing complex interactions among design parameters and resulting outcomes that may be challenging to isolate experimentally.^3,5,7,12,17–20^ While computational methods offer speed, flexibility, and the ability to explore implant performance under diverse motion conditions, some existing tools still demand advanced finite element expertise and/or rely on simplified kinematic assumptions that may fail to capture the complexity of glenohumeral motion.

A standardized method for defining impingement-free ROM is therefore essential in providing a common reference for implant assessment, and a quantitative benchmark for evaluating design-driven improvements.^13,20–25^ The standardized joint coordinate systems recommended by Wu et al.^23^ highlight the level of precision required to accurately model upper-extremity motion, particularly at the glenohumeral joint, where the sequence of humeral rotations with respect to the scapular is crucial.^23^ Alternative definitions are not recommended for the glenohumeral joint as they can yield inconsistent outcomes when rotation order or reference frame assumptions vary. Although ISB prescribes rotation sequence conventions for shoulder motion, global coordinate descriptions can also be applied. This is well elucidated by Rab who showed that the ISB standard and global approaches are mathematically equivalent, with the latter offering more intuitive visualization.^22^ Nevertheless, describing humeral motion relative to the scapula using a Euler rotation sequence remains the most rigorous framework for quantitative motion analysis.

Building on these standardized definitions, the present work presents a CAD-to-Simulink computational pipeline that performs comprehensive, standardized elevation motion sweeps across multiple planes using ISB recommended coordinate conventions.^23^ While conventional CAD or mesh-based approaches can approximate impingement positions, they typically require manual adjustment and simplified assumptions that limit accuracy and reproducibility. Analogous to advanced biomechanical and robotic simulations.^26,27^ The Simscape Multibody toolbox within Simulink was selected for its ability to automatically prescribe humeral positions and rotations according to ISB standards, execute multi-plane motion sweeps, and detect impingement directly from anatomical geometry; capabilities that can be challenging to achieve with conventional methods. The developed framework automatically detects and records impingement positions, providing a geometry-driven, reproducible prediction of impingement-free ROM. By directly integrating CAD geometry into a motion-based simulation environment, this framework offers a robust, Finite Element Method (FEM)-independent platform for preclinical implant assessment and design optimization.

## Methods

The pipeline integrates SolidWorks^®^ (Dassault Systèmes, France) and MATLAB^®^ Simulink Simscape Multibody™ (MathWorks, USA). The workflow proceeds in three stages: (1) model preparation and export from SolidWorks, (2) assembly import and reconstruction within MATLAB, and (3) motion prescription and impingement detection in Simscape Multibody.

### Model preparation

An RTSA assembly model was constructed in SolidWorks by virtually implanting standard generic components (glenosphere, cup, and stem), representing a contemporary Grammont-style design, into scapula and humerus geometries representative of a 50th percentile male anatomy. Implant positioning was performed according to established RTSA surgical guidelines and aligned with clinically reported implantation configurations described in the literature.

**Figure 1.**
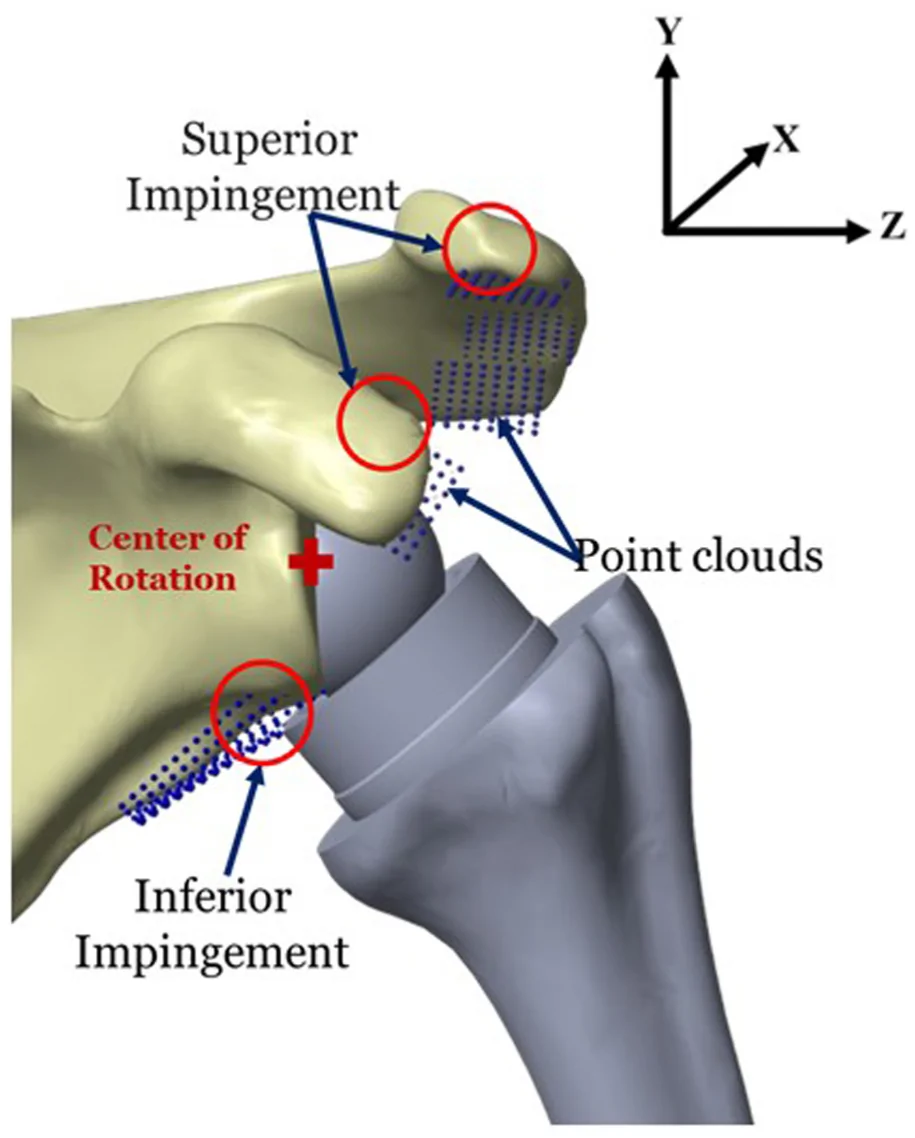
Seeded point clouds used for impingement detection. Grid points were placed at three locations on the acromion and one at the coracoid to detect superior impingement across planes of elevation. A semi-conical distribution along the scapular neck’s lateral border guided detection of inferior impingement.

The final assembly was positioned to represent a clinically realistic position and configuration. Local coordinate systems were aligned to ISB guidelines (Figure 1), ensuring consistent humerus-to-scapula reference frames and correct anatomical orientation.^23^

The assembled model was then exported to Simulink using the Simscape™ Multibody™ Link plugin, generating a STEP (.stp) file for each component and an XML file containing mate definitions, color data, and reference frame information. These files formed the basis for reconstructing the full assembly in Simscape.

### Simscape model reconstruction

The exported XML and STEP files were imported into MATLAB Simscape Multibody, where each anatomical and implant component represented a rigid body block. This process preserved the geometry, co-ordinate frames, and mates of each component. The resulting block-based representation aided reconstruction of the musculoskeletal—implant system as a network of rigid bodies connected by defined joints. A gimbal joint was assigned at the glenohumeral interface to allow three independent rotations about anatomical axes. During import, minor corrections were required where certain SolidWorks mates were not directly transferable due to unsupported or differently interpreted constraints within the Simscape Multibody environment. Coordinate alignment and anatomical correctness were verified using visualization blocks within the Mechanics Explorer visualization tool. This standardized representation retained implant positioning established in SolidWorks while allowing controlled manipulation of kinematics and implant alignment within Simscape.

### Motion prescription

Motions were prescribed following using an iterative MATLAB script. The script defined both the planes of elevation (−60° to 60° in 30° increments) and the discrete elevation angles (−30° to 120° in 1° increments). Each plane of elevation (POE) was set through a rigid transform Simulink block that defined the plane in which humeral elevation would occur. For each POE, the script advanced the humerus through all the prescribed elevation angles sequentially, executing a full motion sweep in each plane before advancing to the next POE. All motions originated from the anatomical neutral position (arm at side), with the scapular held fixed. This automated procedure ensured consistent and reproducible motion definition across all test conditions.

### Impingement detection

Impingement detection was performed using the Simscape Spatial Contact Force blocks which sensed contact and calculated the penetration depth between geometries. Given the complex geometry of the scapula, point clouds were used to increase impingement detection sensitivity as shown in Figure 1.

These point clouds were seeded (2 mm spacing) along known potential impingement regions. Three anchor points were chosen on the acromion (anterior, mid, posterior), one on the coracoid process (front border), and along the inferior lateral border of the scapular as shown in Figure 1.

### Impingement detection

Each point in the seeded cloud was modeled as an independent sensor connected to the Spatial Contact Force block in Simulink. The Spatial Contact Force block generated two outputs: (1) penetration depth data for each seeded point, and (2) a binary contact signal (0 = no contact detected, 1 = contact detected). These two outputs along with the positional data, were streamed automatically into the MATLAB workspace for post-processing and subsequent impingement prediction. Impingement was defined as the elevation angle at which the maximum summed penetration depth across all seeded points exceeded 1 mm for both superior and inferior impingement. This criterion was applied consistently across all planes of elevation. A sensitivity analysis was performed to ensure that variations in point cloud density and anchor placement did not affect impingement detection.

### Neck-shaft angle variation

Additional models representing 145° and 135° neck-shaft configurations were assembled in SolidWorks maintaining the same glenosphere position, cup depth, and component alignment. The assemblies were exported to Simulink following the same workflow as the 155° model.

Impingement detection parameters, point cloud spacing, and motion prescription were held constantly across all configurations to ensure comparability.

### Experimental validation

**Figure 2.**
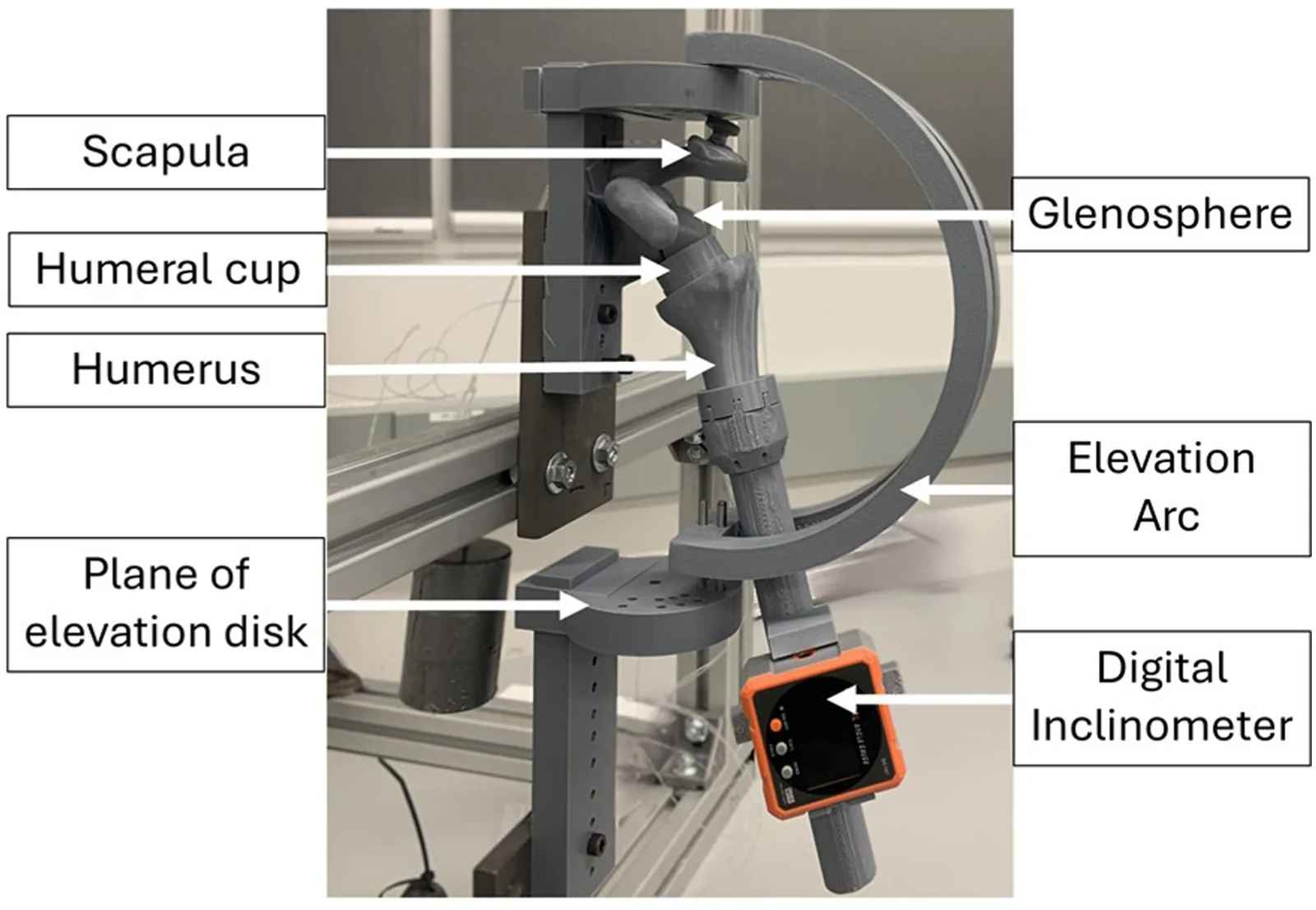
Experimental setup for impingement detection. 3D-printed bone and implant monoblocs were mounted on aluminum extrusions. A 3D-printed elevation arc, attached to a disk with holes marking planes of elevation, guided the humerus along predefined planes. A digital inclinometer mounted on the humerus recorded the angles at which impingement occurred.

Experimental validation was conducted for the 155° neck-angle configuration, which served as the reference model for computational verification. To validate the computational impingement predictions, monobloc models of the humerus and scapula with implanted RTSA components were additive manufactured in PLA. The printed parts were derived directly from the CAD geometries used in the computational model to ensure geometric consistency. The assembly was mounted on a custom testing rig replicating the simulated joint alignment (Figure 2). During testing, the humeral component was manually elevated while maintaining a constant 15 N compressive load to simulate deltoid tension. This load was applied via drop weights attached to the humeral shaft using fishing wire, similar to the setup used in previous studies.^28,29^

Impingement was recorded at the point where elevation ceased using a digital inclinometer, which has a manufacturer-specified accuracy of ±0.2° and a resolution of 0.05°, and was confirmed visually. The plane of elevation was controlled using an adjustable arc, and tests were conducted in five planes (−60° to +60° in 30° increments) to match the computational setup. Each motion was repeated three times per plane, and the elevation angle was recorded for comparison with simulation results.

## Results

**Figure 3.**
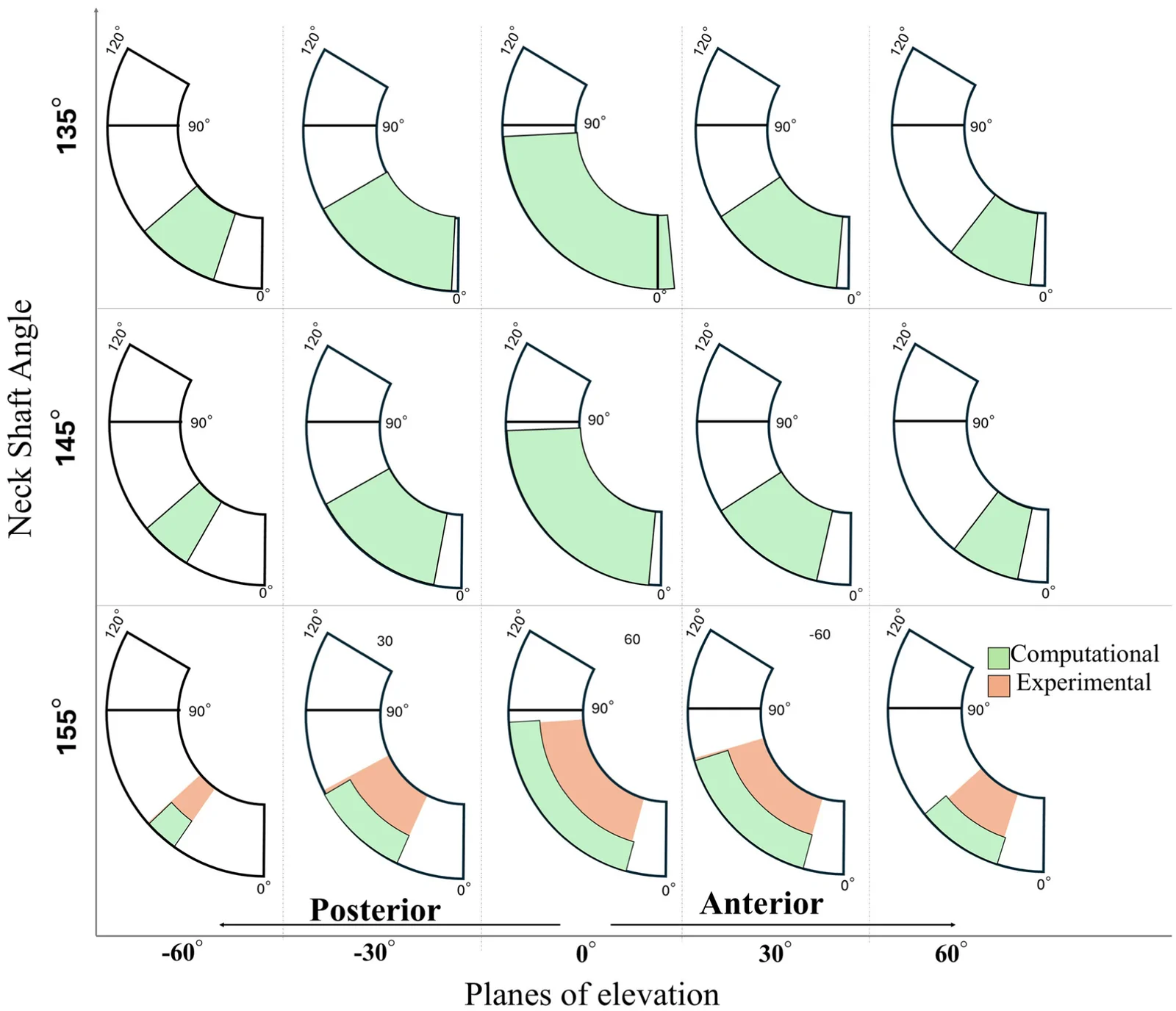
Computational predictions of impingement-free range of motion for neck-shaft angles of 155°, 145°, and 135° across planes of elevation from −60° to 60° in 30° increments. Locations of superior and inferior impingement are indicated, with experimental validation shown for the 155° configuration.

Range of motion was evaluated across the three neck-shaft angles (155°, 145°, and 135°) using the CAD-to-Simulink computational framework. A clear increase in impingement-free motion was observed as the neck-shaft angle decreased (Table 1, Figure 3). In the scapular plane, the 135° configuration exhibited the greatest allowable motion, with inferior impingement occurring at −6° of elevation, compared to 4° for 145° and 14° for 155°, representing an improvement of approximately 20° in adduction.

**Table 1.** Computed superior and inferior impingement elevation angles for varying neck-shaft angles, with experimental results provided in parenthesis for the 155° configuration.

Neck shaft angle	155°					145°					135°					
Plane of elevation	−60°	−30°	0°	30°	60°	−60°	−30°	0°	30°	60°	−60°	−30°	0°	30°	60°
Inferior impingement	38 (38)	24 (24)	14 (13.8)	12 (12)	17 (16.8)	28	10	4	6	10	16	1	−6	−4	5
Superior impingement	45 (45.3)	59 (60)	85 (84.5)	70 (71.3)	48 (46)	47	59	87	55	36	47	59	87	55	36
Impingement-free ROM	7 (7.3)	35 (36)	71 (70.3)	58 (59.3)	31 (29.2)	19	49	83	49	26	31	58	93	59	31

The mean impingement-free ROM increased progressively with decreasing neck–shaft angle, with values of 40.4° (155°), 45.2° (145°), and 54.4° (135°), demonstrating a systematic expansion in allowable motion across design changes. A slight, non-significant delay in superior impingement was observed as the neck-shaft angle decreased, with mean values shifting from 61.4° for 155° to 56.8° for both 145° and 135°, indicating minimal influence on abduction.

Experimental validation of the 155° configuration showed strong agreement with computational predictions, with superior impingement occurring at 61.4 ± 16.8° (experimental) versus 61.4 ± 16.5° (computational) and inferior impingement at 20.9 ± 10.6° versus 21.0 ± 10.6°, respectively. This strong correspondence supports the reliability of the CAD-to-Simulink framework in predicting impingement behavior.

Overall, reducing the neck-shaft angle from 155° to 135° increased impingement-free range of motion across all evaluated planes. Compared to the 155° configuration, the 135° configuration increased ROM by 23° at −30° POE and 22° at 0° POE, with the greatest improvements associated with a delayed onset of inferior impingement.

### Sensitivity analysis

**Figure 4.**
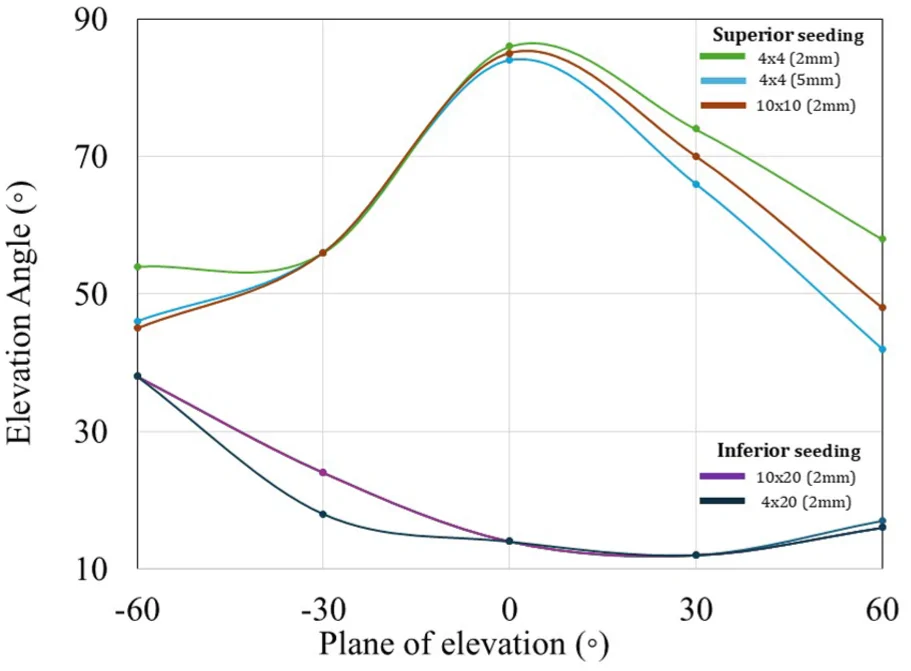
Sensitivity analysis for the 155° neck-shaft angle, showing how point-cloud seeding location along the acromion, coracoid, and inferior lateral border of the scapular neck, as well as seeding density, affect superior and inferior impingement detection across five planes of elevation.

Point-cloud seeding along the acromial and coracoid borders was the primary determinant of impingement detection accuracy (Figure 4). Superior impingement was sensitive to point spacing but not the total number of points. A 4 × 4 point grid with 5 mm spacing produced comparable results to a 10 × 10 point grid with finer spacing (2 mm). Inferior impingement was largely insensitive to seeding, except at the most posterior and anterior planes, where sparser distributions increased variability modestly (∼3 to 7°).

## Discussion

To our knowledge, this is the first study to introduce and validate a CAD-to-Simulink computational pipeline for assessing impingement-free ROM in RTSA. Unlike conventional methods that rely on simplified geometric assumptions, such as Boolean subtractions, mesh overlaps, or clearance thresholds, the proposed framework detects impingement through direct spatial contact between geometries using high resolution point clouds. This geometry driven approach eliminates the need for computationally expensive tools while capturing true surface interactions via point clouds for spatial precision. Notably, while many existing approaches detect collisions only after impingement has occurred, the present proximity-based method identifies early contact onset, allowing a more conservative and physiologically relevant estimate of impingement-free ROM.

Validation against experimental data confirmed the accuracy of the pipeline. For the 155° neck-shaft angle configuration, the mean computational superior and inferior impingement angles were 61.4 ± 16.5° and 21.0 ± 10.6°, respectively, compared to experimental measurements of 61.4 ± 16.8° (superior) and 20.9 ± 10.6° (inferior). Both computational and experimental results were consistent in most positions, with discrepancies likely due to minor misalignments of motion axes and additive manufacturing tolerances. Additionally, experimental measurements are continuous, whereas the computational pipeline evaluates discrete 1° elevation increments, explaining minor observed differences shown in Table 1. Despite these limitations, the results support the framework’s ability to reliably detect impingement.

To assess the framework’s responsiveness to geometric variations, neck-shaft angles of 155°, 145°, and 135° were examined. Decreasing the neck-shaft angle progressively increased impingement-free ROM, primarily driven by delayed inferior impingement during adduction (−6 ± 8°, 4 ± 8°, 21 ± 9° for 135°, 145°, and 155°, respectively), while superior impingement remained relatively constant (Figure 3). These trends align with previously reported biomechanical and computational findings on the effects of implant configuration on impingement-free ROM,^16,30^ confirming that the framework captures realistic, plane-dependent kinematic behavior. Testing of these angles was intended to verify that this method of detection impingement can resolve subtle geometric and positional effects, demonstrating its suitability for robust parametric evaluation.

The accuracy of impingement detection was further evaluated in a sensitivity study, which highlighted the influence of point-cloud seeding locations, spacing and seed density, particularly along the acromion and coracoid borders. Superior impingement predictions were sensitive to point spacing rather than total point number (Figure 4). A 4 × 4 grid with 5 mm spacing produced results comparable to a 10 × 10 grid with 2 mm spacing due to similar coverage of regions prone to impingement. In contrast, a 4 × 4 grid with 2 mm spacing overestimated impingement onset, reflecting delayed detection caused by a concentration of the seeded points at one location. Despite this, superior impingement in the posterior plane (−30°) remained stable across different seeding variations, as humeral contact occurs over a broad acromial area, enhancing the likelihood of accurate impingement detection. Inferior impingement, on the other hand, was largely insensitive to seeding configuration, provided the lateral border of the scapular neck was adequately covered. This was observed when the 10 × 20 point lateral border configuration was reduced to a 4 × 20 configuration, leaving the outer regions with a sparser, resulting in a minor difference of 6°. These results indicate that ensuring adequate point cloud coverage of key anatomical features is more crucial than point density for reproducible impingement detection across different geometries.

RTSA impingement is often assessed computationally using mesh intersection or clearance-based proximity thresholds.^16,20,30^ While these methods can identify general motion trends, they often depend on arbitrary clearance values or coarse geometric resolution, leading to distorted or inconsistent ROM predictions. The current framework overcomes these limitations by coupling CAD-based implant and bone geometries directly with a multibody simulation, where motion is defined and impingement detected. This geometry-driven approach eliminates the need for complex meshing or finite element preprocessing while ensuring accurate, surface interaction.

Furthermore, the proposed framework enables rapid parametric evaluation of implant design variables under consistent motion trajectories. Although initial configuration requires CAD assembly and Simscape Multibody modeling, subsequent motion sweeps and impingement detection are fully automated without further user intervention. Once the pipeline is established, additional configurations can be evaluated in under 5 min per case, with runtime governed by the prescribed simulation time step (2 s per motion increment in this study). Because the workflow operates independently of mesh resolution, it remains computationally efficient, reproducible, and well suited for early-stage design optimization or preclinical screening of new implant geometries. By employing standardized coordinate definitions, results can be directly compared across studies, contributing to the development of a consistent benchmark for computational evaluation of functional ROM in RTSA.

## Limitations and conclusions

While the framework effectively captures geometry and plane dependent effects and aligns closely with experimental measurements, various limitations should be noted. Mean impingement-free ROM values were computed as an equal-weight average across five planes of elevation. Although the scapular plane is often considered the most clinically relevant, the framework evaluates multiple planes to capture plane-specific sensitivity to geometric changes rather than prioritizing a single functional plane. The model also simulates quasi-static, unloaded shoulder motion using a gimbal joint that accounts only for rotational degrees of freedom, with scapulothoracic motion not included. Furthermore, a fixed implant position was assumed, and variations in surgical placement (e.g., glenosphere version, inclination, and humeral height) were not explored in this study, but can be investigated within the same framework through the generation of alternative implant configurations.

In addition to these modeling assumptions, validation was performed using additively manufactured monoblocks. As such, these printed constructs cannot perfectly replicate real bone. Intricate anatomical features, such as bony prominences or undercuts, may be lost due to printing resolution, potentially affecting impingement measurements.

Lastly, the neck-shaft-angle analysis was included as a proof-of-concept to demonstrate the sensitivity of the framework to geometric variations, rather than to provide statistically generalizable clinical conclusions. Accordingly, the reported ROM trends should be interpreted as model-specific, and broader clinical inference would require subject-specific analyses.

Despite these simplifications, the pipeline provides a reliable, reproducible platform for assessing implant performance, supporting rapid parametric evaluation and early-stage design optimization. By integrating CAD-based modeling with multibody simulation, this approach establishes a computationally efficient foundation for future patient-specific and dynamic RTSA studies.
